# Blood Glucose Homeostasis is Preserved in Patients with Mild-to-moderate Spinocerebellar Ataxia Type 2

**DOI:** 10.1007/s12311-026-02076-1

**Published:** 2026-08-29

**Authors:** Raúl Aguilera-Rodríguez, Dennis Almaguer-Gotay, Amarilis Álvarez-Sosa, Marilennis Anidos-Machado, Yanelis Silva-Ricardo, Dany Cuello-Almarales, Annelié Estupiñán-Rodríguez, Luis E. Almaguer-Mederos

**Affiliations:** 1 Center for the Investigation and Rehabilitation of Hereditary Ataxias (CIRAH), Holguín, Cuba; 2 Department of Nuclear Medicine, General University Hospital, Holguín, Cuba; 3 Medical Sciences University, Holguín, Cuba; 4 Department of Laboratory Medicine, Clinical Surgical University Hospital, Holguín, Cuba; 5 Department of Neuromuscular Diseases, University College London Queen Square Institute of Neurology, London, UK

**Keywords:** Glucose homeostasis, Insulin resistance, Insulin secretion, Pathophysiology, Spinocerebellar ataxia type 2

## Abstract

Spinocerebellar ataxia type 2 (SCA2) is a neurodegenerative disorder that shows cerebellar glucose hypometabolism, systemic hypermetabolism and weight loss. This study aimed to explore novel molecular mechanisms of disease for SCA2, based on the assessment of blood glucose homeostasis. A case-control and correlational study was conducted in 79 normoglycemic Cuban patients with SCA2 and 83 sex- and age-matched control subjects during a fasting state. A subset of 20 patients with SCA2 and 19 control individuals underwent an oral glucose tolerance test (OGTT). Several indices for assessing blood glucose homeostasis, derived from the fasting state or the OGTT, were included in the study. Fasting glucose levels showed a small increase, whereas the QUICKI index for insulin sensitivity was slightly decreased among patients. Markers of glucose homeostasis derived from the OGTT were no different between patients and controls. Fasting insulin levels, QUICKI, McAuley´s, HOMA2-%S, HOMA2-IR, and HOMA2-%β indices showed weak to moderate correlations with markers of body composition. McAuley´s, TyG, HOMA2-IR, and HOMA2-%β indices correlated with the age at onset, progression rate, or INAS count. Patients with SCA2 with mild-to-moderate ataxia do not have any major alteration in blood glucose homeostasis. Markers of glucose homeostasis associate with body composition and have nominal modifying effects on disease severity and progression rate in patients with SCA2, with McAuley’s index showing effects more consistently. Further studies are needed to describe the changes in blood glucose homeostasis in the different stages of disease, and to delve into its pathophysiological relevance.

**Supplementary Information:**

The online version contains supplementary material available at https://doi.org/10.1007/s12311-026-02076-1.

## Introduction

Blood glucose homeostasis, a tightly regulated process of maintaining blood glucose at a steady-state level, is critical for properly preserving a healthy cellular energy metabolism [1, 2]. This is particularly relevant to the brain, where glucose metabolism is also important for the synthesis of amino acids, methyl donors derived from one-carbon metabolism, glycolipids, glycoproteins, precursors of neurotransmitters and neuromodulators, oxidative stress management, and neuroplasticity [1, 3, 4]. Not surprisingly, therefore, altered brain glucose metabolism was linked to neurodegenerative disorders including polyglutamine diseases (polyQ), such as Huntington´s disease (HD) [5–7], spinocerebellar ataxias (SCA) type 1, 2, 3, 6, 7, and 17 [8–11], and dentatorubral-pallidoluysian atrophy [12].

Further evidence supporting a role for glucose homeostasis in the pathogenesis of polyQ diseases comes from the study of peripheral blood surrogate markers in patients and animal models [13–23]. Interestingly, increased serum insulin levels, abdominal obesity, hepatosteatosis, dyslipidemia, and altered fatty acid pathways suggestive of insulin resistance, were observed in an Ataxin-2 deficient mouse (Sca2^−/−^) [24]. In an additional study, it was shown that Ataxin-2 lentiviral overexpression in the hypothalamus is important for modulation of insulin sensitivity and body weight in *Atxn2* knockout mice [25]. Besides, it was proposed that genetic variants at the chromosomal locus of human *ATXN2* carry a risk for DM [26]. However, no study has been conducted on blood glucose homeostasis and its clinical relevance in patients with SCA2.

Spinocerebellar ataxia type 2 (MIM: 183090) is an autosomal-dominant polyQ disorder that reaches the highest prevalence worldwide in Holguin province, Cuba. Its genetic etiology consists of a dynamic CAG repeat expansion mutation in the first exon of the *ATXN2* gene (cytogenetic band: 12q24.12) [27–29]. *ATXN2* CAG repeat expansions beyond 31 repeats cause the typical SCA2 cerebellar syndrome, while expansions in the range of 27 to 33 repeats increase the disease risk or progression of amyotrophic lateral sclerosis (ALS) [30–32], frontotemporal dementia (FTD) [33, 34], idiopathic levodopa-responsive Parkinson’s disease [35], and progressive supranuclear palsy (PSP) [36].

SCA2 clinical presentation includes gait ataxia, dysmetria, dysdiadochokinesia, and dysarthria, frequently accompanied by decreased tendon reflexes, leg cramps, kinetic or postural tremor, abnormal eye movements with slowed saccades progressing to nuclear ophthalmoplegia, as well as reduced body mass index and survival [37–41]. The highly variable clinical presentation depends heavily on the length of the CAG repeat tract, with longer repeats causing an earlier disease onset and a faster progression [42, 43]. Heritability estimates for the age at onset suggested an important contribution of additional genetic factors to disease severity [44].

The *ATXN2* gene encodes Ataxin-2 protein, a cytoplasmic RNA-binding factor involved in the regulation of RNA metabolism, mRNA translation, endocytosis, cellular growth and stress responses, cytoskeletal dynamics, and the inhibition of the mechanistic target of rapamycin complex 1 (mTORC1) signaling [45–52]. Upon the unstable expansion of its polyQ domain, the Ataxin-2 protein acquires a toxic gain of function, leading to multi-system brain atrophy by promoting protein aggregation, transcriptional dysregulation, calcium signaling dysregulation, autophagy dysfunction, and oxidative stress [40, 45, 53–59].

Although significant progress was achieved in understanding the mechanisms of disease, and promising benefits were obtained in pre-clinical trials based on treatment with antisense oligonucleotides [60, 61] or Cas13 CRISPR effectors [62] against *Atxn2* mRNA, the full elucidation of the molecular processes and events involved in SCA2 pathophysiology is critical for identifying reliable surrogate biomarkers and additional targets for therapy. In this study, we assessed blood glucose homeostasis in a cohort of normoglycemic patients with SCA2 and healthy control subjects, considering their relationship with molecular and clinical findings.

## Patients and Methods

### Study Design

A case-control and correlational study was conducted to assess markers of blood glucose homeostasis at fasting and during OGTT in Cuban patients with clinical and molecular diagnosis of SCA2 who were referred to CIRAH for follow-up clinical assessments and counseling, and healthy control individuals. The Ethics Committee of the Center for the Investigation and Rehabilitation of Hereditary Ataxias approved the study protocol, and a consent form was obtained from patients in accordance with the principles outlined in the Declaration of Helsinki.

An a priori power analysis was conducted to determine the sample size required to detect a clinically meaningful difference in blood glucose homeostasis parameters. The analysis indicated that using a significance level of α = 0.05, and an effect size of d = 0.60, 74 participants per group were needed to achieve a desired power of 95%. The expected effect size was chosen based on previous studies of blood glucose homeostasis in polyglutamine disorders [16, 17, 21, 63]. Calculations were performed in G*Power 3.1.9.7 [64]. We therefore recruited 79 consecutive patients with SCA2 and 83 healthy control subjects to allow for up to 8% attrition. A subset of 20 patients with SCA2 and 19 healthy controls consented to undergo an OGTT. Healthy control subjects were matched for age (± 2 years), sex, and body mass index, and were recruited from the same geographical and ethnic backgrounds as the patients. Only normoglycemic individuals with fasting serum glucose levels between ≥3.9 and < 5.6 mmol/L [65] and without a previous diagnosis of prediabetes or diabetes mellitus or intestinal malabsorption were included in the study. Patients with SCA2 who were confined to a wheelchair or bed (disease stage 3) [66] were not included in the study.

### Clinical and Genetic Assessments

Diagnosis of SCA2 relied on family history and clinical and molecular criteria. Clinical diagnosis was based on the presence of gait ataxia, dysdiadochokinesis, dysarthria, dysmetria, dysphagia, and slow saccades. Information was also obtained regarding sex, age, and disease duration in years from disease onset to the latest examination. Age at onset was defined as the onset of motor impairment, and it was estimated through a retrospective review of patients’ clinical records and interviews with patients and close relatives. The Scale for the Assessment and Rating of Ataxia (SARA score) was used for assessing ataxia severity [67]; this scale varies from zero to 40 points, increasing with ataxia severity. The progression rate of ataxia at presentation was defined as the ratio between the SARA score and disease duration. The INAS count was used for assessing the severity of non-ataxia signs [68]. The disease stage was assessed as previously specified [66], and the CAG repeat length at the *ATXN2* gene was determined as reported [69].

### Body Composition Measurements

A trained researcher quantified weight (expressed in kilograms, kg) and height (expressed in meters, m) following standardized procedures using a mechanical High-Quality Dial Body Scale (Perlong Medical Equipment Co., Ltd., Nanjing, China). The body mass index (BMI) was defined as the ratio between body weight and the square of the body height, and it was expressed in units of kg/m^2^. The waist circumference (expressed in centimeters, cm) was measured using a flexible, non-stretchable tape measure, following a standardized protocol [70].

### Assessment of Blood Glucose Homeostasis

Blood samples were drawn between 08:00 and 10:00 a.m., after overnight fasting, considering known temporal variations of glucose and insulin levels due to diurnal rhythm [71]. This was followed by an OGTT to assess glucose tolerance status and OGTT-related insulin release. During the OGTT, blood samples were collected immediately before (at minute 0) and 30, 60, 90, and 120 min after ingestion of a 75 g oral glucose load in 3–5 min, after an overnight fast. The serum was separated by centrifugation and stored at − 20 °C until use. Glucose and triglyceride serum levels were measured by spectrophotometry on a Hitachi 902 Automatic Analyzer (Roche, Germany) following the manufacturer´s instructions. Serum insulin levels were quantified using the Insulin [^125^I] immunoradiometric assay (IRMA) kit (Izotop #RK-400CT, Hungary). The inter-assay coefficients of variation were less than 5%.

Several indices for the assessment of blood glucose homeostasis, based on serum glucose, insulin, or triglyceride levels, were included in the study. Fasting insulin sensitivity was assessed by QUICKI [72], McAuley index (Mffm/I) [73], and the homeostasis model assessment of sensitivity (HOMA2-%S) [74]. Fasting insulin resistance was evaluated by the Triglyceride-Glucose index (TyG) [75], and the homeostasis model assessment of insulin resistance (HOMA2-IR) [74]. Besides, fasting β-cell function was assessed by the homeostasis model assessment of beta-cell function (HOMA2-%β) [74]. HOMA2 indices were determined using the HOMA Calculator (v2.2.3).

Well-established indices derived from the OGTT were also quantified. The Gutt insulin sensitivity index (ISI_0, 120_) [76], Matsuda insulin sensitivity index (MI) [77], Stumvoll insulin sensitivity index (Stumvoll−ISI) [78], and Stumvoll metabolic clearance rate (Stumvoll-MCR) [78] were included as surrogate markers of insulin sensitivity/resistance. Additionally, the insulinogenic index (IGI) [79], Stumvoll phase 1 (PH1) and 2 (PH2) [78], and the ratio of the area under the curve for insulin from 0 to 120 min to the area under the curve for glucose from 0 to 120 min (AUC-i/g) [77], were used as surrogate markers of insulin secretion. Besides, the product of the insulinogenic and Matsuda indices (IGI x MI), AUC-i/g and Matsuda indices (AUC-i/g x MI), and the ratio of the insulinogenic index and fasting insulin levels (IGI/FI), were included in the study as composite measures mathematically analogous to the disposition index, indicating the ability of β‑cells to compensate for insulin resistance [80–82].

### Statistical Analysis

Descriptive statistics were applied to the quantitative variables under study. The Kolmogorov-Smirnov test was used to test all variables for normal distribution. The χ^2^ test was used to make comparisons for qualitative variables, whereas the Student’s t-test was used to establish comparisons for continuous variables between patients with SCA2 and control subjects. In cases when the homoscedasticity assumption did not hold, the Welch’s t-test was used instead. For comparison purposes between patients with SCA2 and control subjects, the markers included in the study were corrected for age, sex, or BMI by linear regression. Glucose and insulin curves during OGTT were analyzed using a two-way repeated-measures ANOVA followed by the Šidák’s multiple comparisons test. Correlation analyses were done using the Pearson correlation coefficient (*r*). Stepwise multiple linear regression analyses were performed in patients with SCA2 in the search for factors relevant to the age at disease onset, SARA score, progression rate, or INAS count, considering *ATXN2* expanded alleles, disease duration, and markers of blood glucose homeostasis as independent variables. All tests were two-tailed, and statistical significance was defined as *p* < 0.05. The Holm-Bonferroni method was used to correct for multiple hypothesis testing. The SPSS software version 22.0 (SPSS Inc., Chicago, IL, USA) was used for all statistical analyses.

## Results

In light of the evidence of cerebellar glucose hypometabolism in patients with SCA2 [9], increased insulin serum levels in Ataxin-2 deficient mice (Sca2^−/−^) [24], and on the assumption that altered blood glucose homeostasis may occur in patients with SCA2 as a consequence of polyQ-expanded Ataxin-2 due to its partial loss-of-function, we explored several markers of blood glucose homeostasis from fasting state in patients with SCA2, relative to healthy control individuals. Patients in stage 1 (mild ataxia, *n* = 37) or 2 (moderate ataxia, *n* = 42) were similarly represented in the sample. The distributions of sex, age, body weight, waist circumference, and BMI showed no significant differences between patients with SCA2 and control individuals (Table 1).

**Table 1:** Descriptive statistics and mean comparisons for demographic variables and markers of glucose metabolism from the fasting state, stratified by clinical status

	SCA2 (*n* = 79)	Controls (*n* = 83)	χ^2^/t-test (*p*-value)
Number of individuals (men/women)	79 (30/49)	83 (27/56)	0.526 (0.468)
Age (years)	41.44 (9.07)	39.82 (10.07)	1.075 (0.284)
Waist circumference (cm)	87.07 (9.56)	85.93 (10.69)	0.459 (0.647)^a^
Body weight (kg)	66.46 (12.82)	69.52 (13.31)	−1.857 (0.067)^b^
BMI (kg/m^2^)	25.24 (4.35)	25.33 (4.10)	−0.405 (0.686)^a^
Triglycerides (mmol/L)	1.58 (0.99)	1.52 (0.76)	0.277 (0.782)^c^
FG (mmol/L)	4.83 (0.37)	4.66 (0.47)	2.472 (**0.015**)^d^
FI (µUI/mL)	8.57 (4.02)	8.31 (4.06)	1.111 (0.268)^c^
*Insulin sensitivity*			
QUICKI	0.35 (0.02)	0.37 (0.03)	−2.075 (**0.040**)^c^
Mffm/I	7.27 (1.53)	7.31 (1.26)	−0.173 (0.863)^c^
HOMA2-%S	107.51 (44.37)	117.01 (50.02)	−1.639 (0.103)^e^
*Insulin resistance*			
TyG	3.84 (0.29)	3.82 (0.24)	0.073 (0.942)^b^
HOMA2-IR	1.09 (0.49)	1.03 (0.51)	1.654 (0.100)^e^
*Insulin secretion*			
HOMA2-%β	109.50 (38.61)	113.66 (39.81)	−0.363 (0.717)^d^

*BMI*− body mass index; *FG*− fasting glucose levels; *FI*− fasting insulin levels. Data are expressed as means (SD), except for the number of individuals. ^a^ corrected for age; ^b^ corrected for age and sex; ^c^ corrected for BMI; ^d^ corrected for sex; ^e^ corrected for BMI and sex. Nominally significant effects are shown in bold

### Blood Glucose Regulation Remains Intact in Patients with SCA2

Neither fasting triglycerides nor insulin serum levels showed significant differences between patients and controls. Conversely, fasting glucose levels showed a small increase among patients after correction for sex; however, this difference did not remain significant after correction for multiple comparisons (corrected p-value = 0.210). Besides, patients were no different from controls for insulin secretion (based on the HOMA2-%β index), nor for insulin sensitivity/resistance (based on the TyG, Mffm/I, HOMA2-%S, and HOMA2-IR indices). Conversely, the QUICKI index for insulin sensitivity was slightly decreased with nominal significance among patients after correction for BMI, probably because of the increased glucose levels observed in the patients´ group (Table 1). Once again, this difference did not remain significant after correction for multiple comparisons (corrected p-value = 0.520).

A closer examination of the distribution of QUICKI index values across comparison groups, considering QUICKI values of < 0.35 as indicative of possible insulin resistance [83, 84], produced no significant differences between patients and control individuals (χ^2^ = 0.960; *p* = 0.327). Similar results were obtained when comparing additional insulin sensitivity, resistance, and secretion indices according to previously reported cut-off values [73, 85–88] (Table S1).

**Fig. 1:**
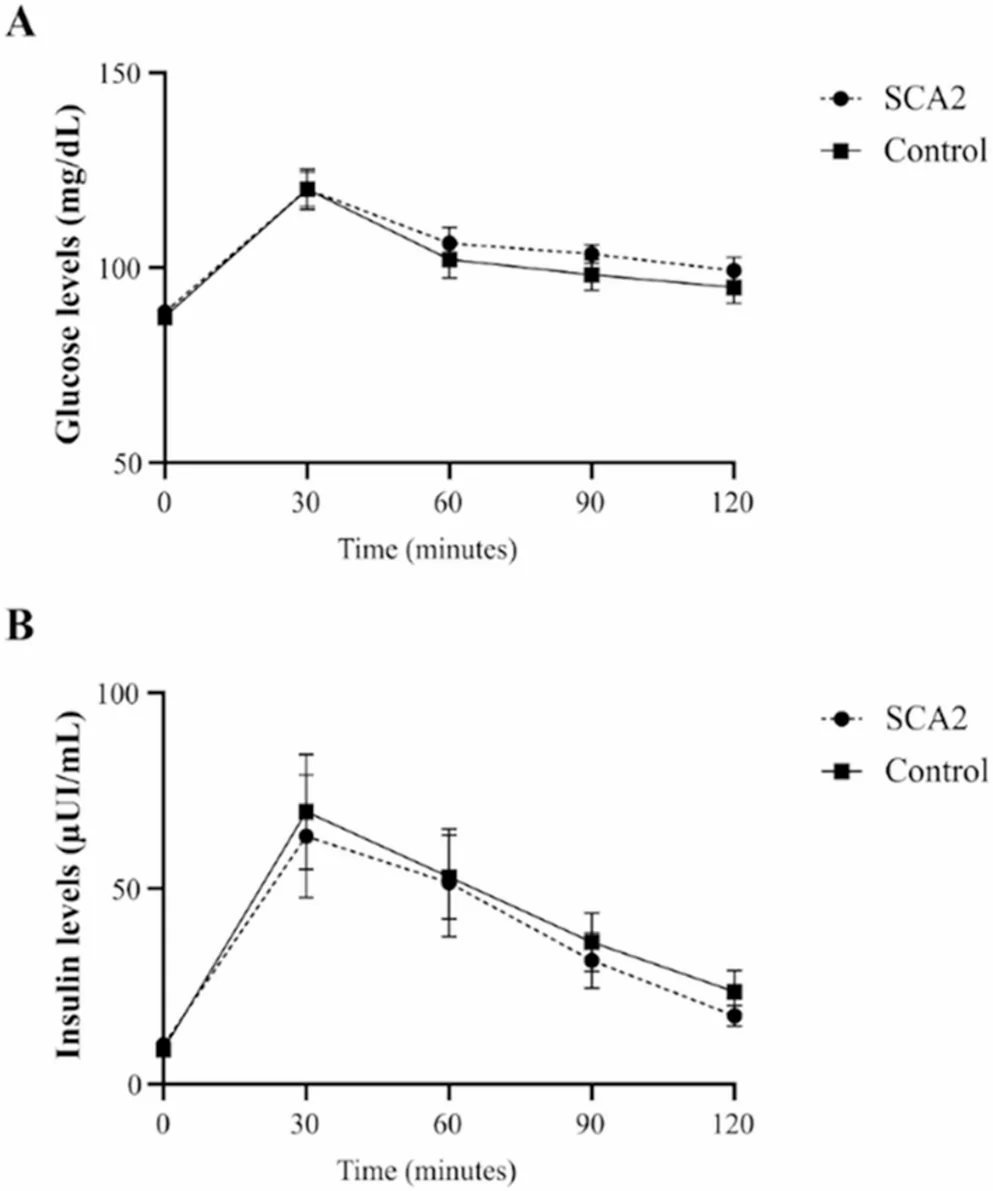
Oral glucose tolerance test (OGTT) in patients with SCA2 (*n* = 20) and control individuals (*n* = 19). **A** Mean serum glucose levels at different time points during OGTT. **B** Mean serum insulin levels at different time points during OGTT. Error bars represent the standard error of the mean

As a further effort to uncover likely abnormalities in blood glucose homeostasis in patients with SCA2, we assessed the response of 20 patients (15 females) and 19 control individuals (13 females) to a glucose load challenge during the OGTT. Comparison groups were no different from each other regarding sex (χ^2^ = 0.201; *p* = 0.654), age (t = 0.467; *p* = 0.644), and BMI (t = 0.302; *p* = 0.765) distributions. Glucose levels during the OGTT were higher in the patients with SCA2, relative to control individuals at every time point, but did not reach statistical significance (Fig. 1A; Table S2). Conversely, insulin levels during the OGTT were lower in the patients with SCA2 relative to control individuals at every time point after the oral glucose load, but no statistical significance was achieved (Fig. 1B; Table S2).

Next, we explored several indices of blood glucose homeostasis derived from the OGTT. All surrogate markers of insulin sensitivity were reduced in the patients relative to control individuals, but none of them reached statistical significance. Similarly, the insulinogenic and Stumvoll phase I (PH1) indices, as surrogate markers of early-phase insulin secretion, and the Stumvoll phase II (PH2) index, as an estimator of the sustained insulin secretion after the initial burst [76], were reduced without significance in the patients. The ratio between the area under the insulin curve and the area under the glucose curve (AUC i/g) as an overall measure of the β‑cell response relative to the glucose challenge [75] also showed a non-significant decrease in the patients. Besides, OGTT-based composite measures as indicators of the ability of β‑cells to compensate for insulin resistance [80–82] showed no significant reductions in the patients with SCA2 relative to control individuals (Table S3).

Altogether, although all insulin sensitivity and secretion indices appeared reduced, and all insulin resistance indices appeared increased in the patients relative to control individuals, only the fasting glucose levels and QUICKI index reached nominal significance. However, these effects did not remain significant after correction for multiple comparisons. Therefore, findings from the fasting state and the OGTT do not consistently support the assumption of altered blood glucose homeostasis in patients with SCA2 with mild-to-moderate ataxia.

### Markers of Blood Glucose Homeostasis Show Weak to Moderate Associations with Body Composition and Disease Severity in Patients with SCA2

Given that disease severity and progression in patients with SCA2 may be influenced by metabolic factors in addition to genetic modifiers [37, 42, 55, 89], we assessed the effects of blood glucose homeostasis markers on anthropometric measures of body composition and disease severity. As expected from established associations between glucose/energy metabolism and body composition [90], fasting insulin levels, QUICKI, McAuley´s, HOMA2-%S, HOMA2-IR, and HOMA2-%β indices showed significant correlations with body weight, waist circumference, and BMI, with more or less diffuse dispersion of data points (Table 2; Figure S1). Most of the associations between markers of glucose homeostasis and waist circumference and BMI, but none of the correlations with body weight, remained significant after correction for multiple hypothesis testing. Conversely, no significant associations were observed between the *ATXN2* CAG repeat length, disease duration, or SARA score, and most of the blood glucose homeostasis markers from the fasting state (Table S4). Comparisons across disease stages did not produce any significant results either (Figure S2). However, McAuley´s index (Mffm/I) for insulin sensitivity showed significant correlations with the age at disease onset (*r*= −0.239; *p* = 0.038), progression rate (*r* = 0.318; *p* = 0.005), and INAS count (*r* = 0.277; *p* = 0.021). Besides, the TyG index for insulin resistance was significantly correlated with the progression rate (*r*= −0.293; *p* = 0.008), and the HOMA2-%β index showed a weak correlation with the INAS count (*r*= −0.241; *p* = 0.046) (Figure S3; Table S4). Nonetheless, only the correlation between McAuley´s index and progression rate remained significant after correction for multiple hypothesis testing (corrected p-value: 0.045).

**Table 2:** Correlation analysis between anthropometric markers of body composition and markers of blood glucose homeostasis in patients with SCA2

	Body weight (kg)	Waist Circ. (cm)	BMI (kg/m^2^)
Triglycerides (mmol/L)	0.207 (0.093)	0.221 (0.073)	0.205 (0.074)
FG (mmol/L)	0.053 (0.671)	0.163 (0.189)	0.035 (0.764)
FI (µUI/mL)	0.268 (**0.033**)	0.334 (**0.007**)	0.295 (**0.011**)
*Insulin sensitivity*			
QUICKI	−0.291 (**0.020**)	−0.351 (**0.004**)	−0.342 (**0.003**)
Mffm/I	−0.279 (**0.026**)	−0.342 (**0.006**)	−0.313 (**0.007**)
HOMA2-%S	−0.215 (0.096)	−0.301 (**0.018**)	−0.283 (**0.017**)
*Insulin resistance*			
TyG	0.158 (0.203)	0.222 (0.071)	0.163 (0.157)
HOMA2-IR	0.262 (**0.037**)	0.333 (**0.007**)	0.294 (**0.011**)
*Insulin secretion*			
HOMA2-%β	0.267 (**0.033**)	0.264 (**0.035**)	0.277 (**0.017**)

*FG*− fasting serum glucose levels; *FI*− fasting serum insulin levels; *TyG*− Triglyceride-Glucose index; *QUICKI*− Quantitative Insulin Sensitivity Check Index; *Mffm/I*− McAuley index; *HOMA2-%β*− Homeostasis Model Assessment of Beta-Cell function; *HOMA2-%S*− Homeostasis Model Assessment of Insulin Sensitivity; *HOMA2-IR*− Homeostasis Model Assessment of Insulin Resistance; *Waist Circ.*− Waist circumference; *BMI*− body mass index. The Pearson´s correlation coefficients (r) and the corresponding *p*-values are shown. Significant associations are shown in bold

As a major modifier of clinical severity, the *ATXN2* CAG repeat length was strongly correlated with the age at onset (*r*= −0.713; *p* < 0.001), SARA score (*r* = 0.311; *p* = 0.006), progression rate (*r* = 0.746; *p* < 0.001), and INAS count (*r* = 0.427; *p* < 0.001). Besides, disease duration was associated with the SARA score (*r* = 0.551; *p* < 0.001). In addition, the BMI was the only anthropometric measure of body composition relevant to disease severity, showing a significant correlation with the age at onset (*r* = 0.264; *p* = 0.024) (Table S5).

Based on these correlations, we re-tested the association between markers of clinical severity and parameters or indices of blood glucose metabolism by including the CAG repeat length, BMI, and/or disease duration as additional predictors in multiple linear regression models. Regression of the CAG repeat length, BMI, and markers of blood glucose metabolism on the age at onset produced no significant results for markers of glucose homeostasis (Table S6). Similarly, no marker of blood glucose metabolism was associated with the SARA score after correction for the CAG repeat length and disease duration (Table S7). Conversely, after correction for the CAG repeat length by multiple linear regression on the progression rate, the McAuley’s index remained significant (β(SE) = 0.016 (0.007); *p* = 0.020), and a new association with the HOMA2-IR index emerged (β(SE) = −0.047 (0.022); *p* = 0.038) (Table S8). No marker of blood glucose metabolism was associated with the INAS count after correction for the CAG repeat length (Table S9). None of the stronger effects obtained during multiple linear regression analysis for the markers of blood glucose metabolism remained significant after correction for multiple hypothesis testing.

Overall, association analyses indicate that blood glucose metabolism associates with body composition and has weak to moderate effects on disease severity and progression rate in patients with SCA2. McAuley’s index of insulin sensitivity and HOMA2-IR were the only parameters showing effects independent of the CAG repeat expansion, with McAuley’s index of insulin sensitivity showing significant nominal effects more consistently.

## Discussion

The clarification of the molecular mechanisms of disease is crucial for identifying reliable surrogate biomarkers and developing effective therapies for SCA2. As the novel contribution of the present study, we now explored surrogate markers of blood glucose homeostasis in patients with SCA2 to identify those relevant to SCA2 pathophysiology.

Evidence supporting a role for blood glucose homeostasis in the pathogenesis of polyglutamine disorders is scarce and conflicting. Reports showing altered glucose homeostasis in mouse models or patients with HD, SCA1, SCA3, SCA7 or SBMA [13, 15–19, 21, 22, 91] were challenged by additional studies showing no difference in plasma glucose levels, insulin sensitivity and secretion between HD patients and control individuals from fasting state or during the OGTT [63, 92]. Similarly, we obtained no convincing evidence for an altered blood glucose homeostasis in patients with mild-to-moderate SCA2, apart from a subtle increase in fasting serum glucose levels along with a decreasing QUICKI index for insulin sensitivity after correction for BMI, which may be indicative of an incipient pro-insulin resistance phenotype [72, 83]. These effects are reminiscent of observations in the Sca2^−/−^ mouse, where increased insulin levels in the pancreas and blood serum were described [24], as occurs in insulin resistance syndromes [93], and might reflect an Ataxin-2 partial loss-of-function due to its polyQ expansion and subsequent aggregation.

Interestingly, pancreatic β-cells, similar to neurons, are long-lived cells that show a very slow replication rate (estimated at 0.1–0.5% in the adult), remain practically quiescent throughout adulthood [94–97], and are highly sensitive to stress granule dysfunction and the aggregation of RNA‑binding proteins [98–100]. Remarkably, evidence indicates that polyQ-expanded HTT protein undergoes aggregation in pancreatic β-cells of mouse models of HD [15, 101, 102], sequestering key insulin signaling pathway factors into the aggregates and disrupting insulin secretion [103, 104]. Conversely, findings from fasting state and during OGTT indicate that patients with SCA2 with mild-to-moderate ataxia do not have any major pancreatic β-cell defect, suggesting that polyQ-expanded Ataxin-2 does not interfere substantially with insulin secretion. This absence of effect may be related to a lack of key insulin signaling factors being sequestered into polyQ-expanded Ataxin-2 aggregates in pancreatic β-cells. Unfortunately, though the accumulation of polyQ-expanded Ataxin-2 protein aggregates is the pathological hallmark in the brains of patients with SCA2 [105, 106], there is no available evidence that any published study has examined the aggregation of polyQ-expanded Ataxin-2 protein in pancreatic β‑cells, which limits the understanding of its potential impacts on insulin synthesis and secretion.

Markers of blood glucose homeostasis seem to be of minor importance for body composition in patients with HD or SCA1 [17, 63], though the limited sample size in these studies may be hiding true effects. Contrary to those findings in HD or SCA1 patients, a direct correlation between HOMA-IR and BMI was reported in a larger cohort of patients with SBMA [91]. Similarly, most markers of blood glucose homeostasis were significantly associated with body weight, waist circumference, and BMI in our SCA2 cohort with mild-to-moderate ataxia. Given that Ataxin-2 protein has been involved in lipid storage and energy metabolism [24, 37, 54, 56, 107–113], the observed correlations suggest a conserved functional interplay between blood glucose homeostasis and body composition, to cope with the metabolic stress and systemic energy deficits posed by the continuous expression of polyQ-expanded Ataxin-2 in peripheral tissues involved in energy storage.

Findings of brain glucose homeostasis problems usually correlate with markers of pathology or disease severity in common neurodegenerative diseases [5, 114], and also in rare polyglutamine disorders [13, 16, 115–117]. However, these correlations seem to be elusive when it comes to peripheral markers of blood glucose homeostasis in polyglutamine disorders. Indeed, the CAG repeat length showed a significant correlation only with the acute insulin response in HD patients, but not with additional markers of glucose homeostasis in patients with HD, SCA1, SCA3, or SBMA [16, 17, 21, 63, 91]. Besides, markers of blood glucose homeostasis appear to possess negligible significance for severity or duration of neurological manifestations in patients with HD, SCA1, or SBMA [17, 63, 91, 92], though a direct association between insulin levels and age at onset was reported in SCA3 patients [21]. Likewise, we found no strong association between the CAG repeat at *ATXN2* expanded alleles, SARA score, or disease duration, and markers of blood glucose homeostasis in Cuban patients with SCA2. Besides, we obtained no evidence for meaningful changes in glucose homeostasis across disease stages. These findings may reflect a true absence of biological effects, or may be due to the narrow range of expansions described in this study (the largest allele with 48 repeats, and those with 32 to 40 repeats representing >80% of the total distribution). Remarkably, results from studies in animal models for HD or SCA7 suggest that only very large CAG repeat expansions can cause impaired blood glucose homeostasis and even symptoms of diabetes *mellitus* [15, 22, 118]. Further investigations of blood glucose homeostasis in infantile/juvenile patients with SCA2 with much longer CAG repeats might help to tackle this issue.

As a crucial novel finding, in this cohort of individuals with mild-to-moderate SCA2, we observed an unexpected pattern in which higher estimated peripheral insulin sensitivity (Mffm/I) and lower insulin resistance (TyG, HOMA2-IR) were associated with greater disease worsening, alongside an inverse association between HOMA2-%β for pancreatic β-cell function and the INAS count. At first glance, these findings appear counterintuitive given the established links between metabolic dysfunction and neurodegeneration [119, 120]. However, accumulating evidence in Huntington’s disease suggests that systemic metabolic alterations evolve dynamically across disease stages and may substantially decouple fasting-derived insulin indices from underlying pathophysiology. Huntington’s disease is characterized by early and progressive disturbances in energy balance, including hypermetabolism, weight loss, and reduced adiposity, which can occur even in clinically mild stages [121, 122]. These changes are accompanied by alterations in lipid metabolism, insulin secretion, and endocrine regulation, with reports of impaired insulin secretion and variable effects on insulin sensitivity in clinical cohorts [16, 63, 123]. Importantly, fasting-based surrogate indices of insulin sensitivity and resistance are highly dependent on circulating insulin and triglyceride concentrations; therefore, reductions in insulin secretion (as reflected by lower HOMA2-%β) and decreases in lipid availability may artifactually increase estimates of insulin sensitivity and reduce measures of insulin resistance, even in the context of ongoing neurodegeneration.

This interpretation is consistent with prior reports of heterogeneous and stage-dependent metabolic phenotypes in Huntington’s disease, where fasting insulin indices may remain normal or even suggest improved insulin sensitivity despite abnormal dynamic insulin responses and progressive clinical decline [63, 123]. Similar dissociations between peripheral metabolic indices and disease severity have also been described across other neurodegenerative disorders, including Alzheimer’s disease and amyotrophic lateral sclerosis, where weight loss, hypermetabolism, and systemic energy imbalance contribute to paradoxical changes in fasting insulin-derived measures [124–126]. Collectively, these findings support a model in which peripheral insulin-related indices in polyglutamine disorders reflect systemic metabolic adaptation rather than direct mechanistic drivers of neurological progression. Consequently, caution is warranted when interpreting fasting-derived insulin sensitivity and resistance markers as mediators of disease severity and progression in SCA2.

Notably, McAuley´s index was the blood glucose homeostasis marker more consistently associated with clinical severity. Given that McAuley´s index captures lipid-driven insulin sensitivity [73], and the confirmed involvement of altered lipid metabolism in SCA2 [56, 112], its association with markers of clinical severity might reflect effects of lipids rather than effects of insulin levels. In light of recent progress on blood-based biomarker discovery for neurodegenerative diseases [127], it might be important to address the potential relevance of plasma lipids as candidate biomarkers in patients with SCA2.

Regarding the limitations and future extension of this study, it is acknowledged that the inclusion of patients in disease stages 1 or 2 only, constrains the full assessment of the effects of variations in blood glucose homeostasis on disease severity. Cross-sectional studies including presymptomatic individuals and patients in disease stage 3, along with longitudinal studies, might be very helpful for understanding the dynamic changes in blood glucose homeostasis linked to polyQ-expanded Ataxin-2 protein expression. Besides, several factors influencing glucose homeostasis, including dietary food patterns, physical activity, sleep quality, and smoking habits, among others [128, 129], were not assessed in this study. In addition, since the progression rate in SCA2 follows a non-linear trajectory [130] and should be assessed longitudinally, the ratio between the SARA score and disease duration in our study reflects the disease progression rate at presentation only. As a result, further studies should be undertaken to clarify the significance of these factors on the findings reported here.

## Conclusions

Altogether, this study indicates that patients with SCA2 with mild-to-moderate ataxia do not have any major alterations in blood glucose homeostasis; nonetheless, it does not exclude the possibility that glucose homeostasis problems may occur in patients with SCA2 with severe ataxia or larger repeat lengths. In addition, normal variation in markers of glucose homeostasis is associated with body composition and has weak-to-moderate modifying effects on disease severity and progression in patients with SCA2, with McAuley’s index showing more consistent effects. Further longitudinal studies, from pre-symptomatic to advanced disease stages, are needed to clearly describe the dynamic changes in blood glucose homeostasis across different disease stages and to further define its mechanistic relevance in SCA2 pathophysiology.

## Supplementary Information

Below is the link to the electronic supplementary material.

ESM 1 (DOCX 1.17 MB)

## Data Availability

The datasets used during the current study are available from the corresponding author on reasonable request.
