# Supplementary material for: Blood Glucose Homeostasis is Preserved in Patients with Mild-to-moderate Spinocerebellar Ataxia Type 2
Source: Cerebellum. 2026 Aug 29;25(5):135. doi: 10.1007/s12311-026-02076-1 (PMC13526137; doi:10.1007/s12311-026-02076-1)
Supplement: Supplementary file 1 [file 12311_2026_2076_MOESM1_ESM.docx]

***Supplementary***

**Fig. S1.** Scatter plots for the linear regression of markers of glucose homeostasis on anthropometric measures of body composition in patients with SCA2. FI− fasting serum insulin levels; QUICKI− Quantitative Insulin Sensitivity Check Index; Mffm/I− McAuley index; HOMA2-%S− Homeostasis Model Assessment of Insulin Sensitivity; HOMA2-IR− Homeostasis Model Assessment of Insulin Resistance; HOMA2-%β− Homeostasis Model Assessment of Beta-Cell function. R^2^ – Coefficient of determination. None of the associations was significant.

**Fig. S2.** Comparison of markers of glucose homeostasis across disease stages in patients with SCA2. FG− fasting serum glucose levels; FI− fasting serum insulin levels; QUICKI− Quantitative Insulin Sensitivity Check Index; Mffm/I− McAuley index; HOMA2-%S− Homeostasis Model Assessment of Insulin Sensitivity; TyG− Triglyceride-Glucose index; HOMA2-IR− Homeostasis Model Assessment of Insulin Resistance; HOMA2-%β− Homeostasis Model Assessment of Beta-Cell function.

**Fig. S3.** Scatter plots for the linear regression of indices of glucose homeostasis on markers of clinical severity in patients with SCA2. FG− fasting serum glucose levels; FI− fasting serum insulin levels; QUICKI− Quantitative Insulin Sensitivity Check Index; Mffm/I− McAuley index; HOMA2-%S− Homeostasis Model Assessment of Insulin Sensitivity; TyG− Triglyceride-Glucose index; HOMA2-IR− Homeostasis Model Assessment of Insulin Resistance; HOMA2-%β− Homeostasis Model Assessment of Beta-Cell function. R^2^ – Coefficient of determination.

**Table S1.** Comparisons between patients with SCA2 and control individuals for indices of blood glucose homeostasis according to pre-defined cut-off values indicative of insulin resistance.

|  | SCA2  n (%) | Controls  n (%) | χ^2^  (p-value) |
| --- | --- | --- | --- |
| *Insulin sensitivity* |  |  |  |
| QUICKI (<0.35) | 23 (29.11) | 19 (22.89) | 0.816 (0.366) |
| Mffm/I (≤5.0) | 4 (5.06) | 2 (2.41) | 0.228 (0.633)^a^ |
| HOMA2-%S (<60) | 6 (7.59) | 8 (10.96) | 0.214 (0.644) |
| *Insulin resistance* |  |  |  |
| TyG (≥4.50) | 2 (2.53) | 1 (1.20) | 0.002 (0.966)^a^ |
| HOMA2-IR (≥1.40) | 14 (17.72) | 13 (15.66) | 0.124 (0.725) |
| *Insulin secretion* |  |  |  |
| HOMA2-%β (<60) | 3 (3.79) | 2 (2.41) | 0.003 (0.955)^a^ |

QUICKI− Quantitative Insulin Sensitivity Check Index; Mffm/I− McAuley index; HOMA2-%S− Homeostasis Model Assessment of Insulin Sensitivity; TyG− Triglyceride-Glucose index; HOMA2-IR− Homeostasis Model Assessment of Insulin Resistance; HOMA2-%β− Homeostasis Model Assessment of Beta-Cell function. χ^2^ − chi-square test; ^a^ – with Yate´s correction for continuity.

**Table S2.** Mean comparisons between patients with SCA2 and control individuals for the serum glucose and insulin levels during the oral glucose tolerance test.

|  | SCA2 | Controls | Mean difference | Adjusted p-value^♦^ |
| --- | --- | --- | --- | --- |
| Serum glucose (mg/dL) |  |  |  |  |
| *Fasting* | 88.66 (5.01) | 87.45 (8.62) | 1.213 | 0.989 |
| *30-min post-OGTT* | 120.17 (23.46) | 120.16 (19.55) | 0.002 | 0.999 |
| *60-min post-OGTT* | 106.29 (18.20) | 102.14 (20.76) | 4.153 | 0.972 |
| *90-min post-OGTT* | 103.55 (10.72) | 98.25 (17.21) | 5.303 | 0.778 |
| *120-min post-OGTT* | 99.28 (15.28) | 94.93 (17.98) | 4.348 | 0.936 |
| Serum insulin (µUI/mL) |  |  |  |  |
| *Fasting* | 10.01 (6.05) | 8.89 (3.85) | 1.118 | 0.958 |
| *30-min post-OGTT* | 63.34 (23.64) | 69.63 (24.04) | -6.293 | 0.999 |
| *60-min post-OGTT* | 51.47 (28.63) | 52.98 (26.87) | -1.507 | 0.999 |
| *90-min post-OGTT* | 31.67 (14.97) | 36.29 (12.42) | -4.623 | 0.995 |
| *120-min post-OGTT* | 17.47 (9.36) | 23.66 (13.67) | -6.186 | 0.850 |

OGTT− Oral glucose tolerance test. Data are expressed as means (SD). ^♦^− Resulting from Šidák's multiple comparisons test for five comparisons during two-way repeated-measures ANOVA.

**Table S3.** Descriptive statistics and mean comparisons for blood glucose metabolism indices derived from the oral glucose tolerance test, stratified by clinical status.

| Indices | SCA2 | Controls | t-test (p-value) |
| --- | --- | --- | --- |
| *Insulin sensitivity* |  |  |  |
| Gutt ISI_0,120_ (GI) | 55.62 (33.59) | 73.18 (23.55) | 1.619 (0.119) |
| Matsuda ISI (MI) | 9.041 (4.293) | 9.888 (3.916) | 0.593 (0.558) |
| Stumvoll ISI (SI) | 0.112 (0.017) | 0.114 (0.015) | 0.328 (0.745) |
| Stumvoll MCR | 9.471 (1.432) | 9.597 (1.237) | 0.279 (0.782) |
| *Insulin secretion* |  |  |  |
| Insulinogenic index (IGI) | 0.559 (0.431) | 0.691 (0.558) | 0.685 (0.500) |
| Stumvoll PH1 | 823.20 (218.4) | 848.2 (270.5) | 0.273 (0.787) |
| Stumvoll PH2 | 222.00 (49.79) | 226.70 (57.73) | 0.239 (0.813) |
| AUC-i/g | 0.184 (0.067) | 0.247 (0.124) | 1.595 (0.131) |
| *Composite* |  |  |  |
| IGI x MI | 4.598 (2.981) | 6.735 (4.827) | 1.478 (0.152) |
| AUC-i/g x MI | 1.835 (0.702) | 2.095 (0.845) | 0.896 (0.380) |
| IGI / FI | 0.112 (0.108) | 0.115 (0.084) | 0.082 (0.936) |

Gutt ISI 0,120 (GI)– Gutt Insulin Sensitivity Index; Matsuda ISI (MI) – Matsuda insulin sensitivity index; Stumvoll ISI– Stumvoll insulin sensitivity index; Stumvoll MCR– Stumvoll metabolic clearance rate; Stumvoll PH1– Stumvoll first-phase insulin secretion index; Stumvoll PH2– Stumvoll second-phase insulin secretion index; AUC-i/g– ratio between the area under the insulin curve and the area under the glucose curve; FI− fasting insulin levels. Data are expressed as means (SD).

**Table S4.** Correlation analysis between markers disease severity and blood glucose homeostasis parameters/indices in patients with SCA2.

| Parameters/Indices | CAG repeat | AO (years) | SARA score | DD (years) | Progression rate | INAS count |
| --- | --- | --- | --- | --- | --- | --- |
| Triglycerides (mmol/L) | -0.014 (0.902) | 0.025 (0.824) | -0.115 (0.310) | 0.103 (0.364) | -0.149 (0.186) | -0.042 (0.726) |
| FG (mmol/L) | -0.035 (0.760) | 0.081 (0.479) | 0.025 (0.827) | 0.091 (0.420) | -0.033 (0.774) | 0.083 (0.486) |
| FI (µUI/mL) | 0.105 (0.371) | 0.172 (0.137) | -0.122 (0.290) | -0.121 (0.295) | -0.109 (0.345) | -0.179 (0.140) |
| *Insulin sensitivity* |  |  |  |  |  |  |
| QUICKI | -0.055 (0.643) | -0.163 (0.160) | 0.054 (0.644) | 0.037 (0.746) | 0.087 (0.453) | 0.146 (0.230) |
| Mffm/I | 0.188 (0.108) | -0.239 (**0.038**) | 0.191 (0.097) | -0.002 (0.989) | 0.318 (**0.005**) | 0.277 (**0.021**) |
| HOMA2-%S | -0.045 (0.702) | -0.134 (0.247) | 0.021 (0.856) | 0.021 (0.859) | 0.062 (0.591) | 0.156 (0.201) |
| *Insulin resistance* |  |  |  |  |  |  |
| TyG | -0.209 (0.068) | 0.170 (0.133) | -0.177 (0.117) | 0.060 (0.598) | -0.293 (**0.008**) | -0.162 (0.174) |
| HOMA2-IR | 0.104 (0.379) | 0.175 (0.131) | -0.115 (0.319) | -0.110 (0.343) | -0.110 (0.341) | -0.169 (0.166) |
| *Insulin secretion* |  |  |  |  |  |  |
| HOMA2-%β | 0.098 (0.407) | 0.104 (0.371) | -0.109 (0.347) | -0.155 (0.179) | -0.061 (0.597) | -0.241 (**0.046**) |

FG− fasting serum glucose levels; FI− fasting serum insulin levels; TyG− Triglyceride-Glucose index; QUICKI− Quantitative Insulin Sensitivity Check Index; Mffm/I− McAuley index; HOMA2-%β− Homeostasis Model Assessment of Beta-Cell function; HOMA2-%S− Homeostasis Model Assessment of Insulin Sensitivity; HOMA2-IR− Homeostasis Model Assessment of Insulin Resistance; AO− Age at disease onset; DD− Disease duration. The Pearson´s correlation coefficients (*r*) and the corresponding p-values are shown. Significant associations are shown in bold.

**Table S5.** Correlation analysis between markers of body composition and markers of blood glucose homeostasis in patients with SCA2.

| Parameters/Indices | Body weight (kg) | Waist Circ. (cm) | BMI (kg/m^2^) |
| --- | --- | --- | --- |
| CAG repeat | -0.104 (0.415) | -0.051 (0.687) | -0.076 (0.520) |
| AO (yrs) | 0.167 (0.180) | 0.191 (0.124) | 0.264 (**0.024**) |
| SARA score | -0.221 (0.074) | -0.141 (0.257) | -0.187 (0.106) |
| DD (years) | -0.203 (0.100) | -0.239 (0.052) | -0.348 (**0.002**) |
| Progression rate | -0.210 (0.091) | -0.170 (0.173) | -0.200 (0.084) |
| INAS count | -0.205 (0.098) | -0.222 (0.074) | -0.195 (0.109) |

AO− Age at disease onset; DD− Disease duration; Waist Circ.− Waist circumference; BMI− body mass index. The Pearson´s correlation coefficients (*r*) and the corresponding p-values are shown. Significant associations are shown in bold.

**Table S6.** Association of markers of blood glucose homeostasis from fasting state with the age at disease onset in multiple linear regression analysis in patients with SCA2.

| Parameters | Multiple linear model | | | |
| --- | --- | --- | --- | --- |
|  | R^2^_adj_ | Regression coefficient (SE) | p-value |  |
| *ATXN2* expanded alleles | 0.533 | -0.033 (0.004) | **<0.001** |  |
| BMI (kg/m^2^) |  | 0.007 (0.003) | **0.013** |  |
| FG (mmol/L) | 0.530 | 0.020 (0.030) | 0.511 |  |
| FI (µUI/mL) | 0.523 | 0.005 (0.004) | 0.140 |  |
| *Insulin sensitivity* |  |  |  |  |
| QUICKI | 0.512 | -0.510 (0.602) | 0.400 |  |
| Mffm/I | 0.508 | -0.005 (0.008) | 0.579 |  |
| HOMA2-%S | 0.508 | 0.0001 (0.0001) | 0.650 |  |
| *Insulin resistance* |  |  |  |  |
| TyG | 0.527 | 0.010 (0.042) | 0.814 |  |
| HOMA2-IR | 0.524 | 0.044 (0.029) | 0.128 |  |
| *Insulin secretion* |  |  |  |  |
| HOMA2-%β | 0.510 | 0.0001 (0.0001) | 0.462 |  |

FG− fasting serum glucose levels; FI− fasting serum insulin levels; TyG− Triglyceride-Glucose index; QUICKI− Quantitative Insulin Sensitivity Check Index; Mffm/I− McAuley index; HOMA2-%β− Homeostasis Model Assessment of Beta-Cell function; HOMA2-%S− Homeostasis Model Assessment of Insulin Sensitivity; HOMA2-IR− Homeostasis Model Assessment of Insulin Resistance. R^2^_adj_ – Adjusted coefficient of determination. SE – Standard error of the regression coefficient. The multiple linear models were based on the linear regression of the *ATXN2* CAG repeat length and individual glucose homeostasis parameters/indices on the age at disease onset. Significant associations are shown in bold.

**Table S7.** Association of markers of blood glucose homeostasis from fasting state with the SARA score in multiple linear regression analysis in patients with SCA2.

| Parameters | Multiple linear model | | | |
| --- | --- | --- | --- | --- |
|  | R^2^_adj_ | Regression coefficient (SE) | p-value |  |
| *ATXN2* expanded alleles | 0.359 | 0.411 (0.125)  0.366 (0.064) | **0.002**  **<0.001** |  |
| Disease duration (yrs) |  |  |  |  |
| FG (mmol/L) | 0.347 | 0.197 (0.890) | 0.825 |  |
| FI (µUI/mL) | 0.345 | -0.074 (0.104) | 0.477 |  |
| *Insulin sensitivity* |  |  |  |  |
| QUICKI | 0.349 | 11.097 (16.957) | 0.515 |  |
| Mffm/I | 0.369 | 0.376 (0.234) | 0.113 |  |
| HOMA2-%S | 0.347 | 0.005 (0.009) | 0.590 |  |
| *Insulin resistance* |  |  |  |  |
| TyG | 0.369 | -1.963 (1.242) | 0.119 |  |
| HOMA2-IR | 0.353 | -0.749 (0.819) | 0.364 |  |
| *Insulin secretion* |  |  |  |  |
| HOMA2-%β | 0.351 | -0.008 (0.010) | 0.414 |  |

FG− fasting serum glucose levels; FI− fasting serum insulin levels; TyG− Triglyceride-Glucose index; QUICKI− Quantitative Insulin Sensitivity Check Index; Mffm/I− McAuley index; HOMA2-%β− Homeostasis Model Assessment of Beta-Cell function; HOMA2-%S− Homeostasis Model Assessment of Insulin Sensitivity; HOMA2-IR− Homeostasis Model Assessment of Insulin Resistance. R^2^_adj_ – Adjusted coefficient of determination. SE – Standard error of the regression coefficient. The multiple linear models were based on the linear regression of the *ATXN2* CAG repeat length, disease duration, and individual glucose homeostasis parameters/indices on the SARA score. Significant associations are shown in bold.

**Table S8.** Association of markers of blood glucose homeostasis from fasting state with disease progression rate in multiple linear regression analysis in patients with SCA2.

| Parameters | Multiple linear model | | | |
| --- | --- | --- | --- | --- |
|  | R^2^_adj_ | Regression coefficient (SE) | p-value |  |
| *ATXN2* expanded alleles | 0.528 | 0.031 (0.004) | **<0.001** |  |
| FG (mmol/L) | 0.544 | 0.002 (0.027) | 0.949 |  |
| FI (µUI/mL) | 0.521 | -0.005 (0.003) | 0.069 |  |
| *Insulin sensitivity* |  |  |  |  |
| QUICKI | 0.533 | 0.659 (0.491) | 0.184 |  |
| Mffm/I | 0.557 | 0.016 (0.007) | **0.020** |  |
| HOMA2-%S | 0.528 | 0.002 (0.001) | 0.329 |  |
| *Insulin resistance* |  |  |  |  |
| TyG | 0.565 | -0.070 (0.037) | 0.062 |  |
| HOMA2-IR | 0.550 | -0.047 (0.022) | **0.038** |  |
| *Insulin secretion* |  |  |  |  |
| HOMA2-%β | 0.537 | 0.001 (0.001) | 0.127 |  |

FG− fasting serum glucose levels; FI− fasting serum insulin levels; TyG− Triglyceride-Glucose index; QUICKI− Quantitative Insulin Sensitivity Check Index; Mffm/I− McAuley index; HOMA2-%β− Homeostasis Model Assessment of Beta-Cell function; HOMA2-%S− Homeostasis Model Assessment of Insulin Sensitivity; HOMA2-IR− Homeostasis Model Assessment of Insulin Resistance. R^2^_adj_ – Adjusted coefficient of determination. SE – Standard error of the regression coefficient. The multiple linear models were based on the linear regression of the *ATXN2* CAG repeat length and individual glucose homeostasis parameters/indices on the disease progression rate. Significant associations are shown in bold.

**Table S9.** Association of markers of blood glucose homeostasis from fasting state with the INAS count in multiple linear regression analysis in patients with SCA2.

| Parameters | Multiple linear model | | | |
| --- | --- | --- | --- | --- |
|  | R^2^_adj_ | Regression coefficient (SE) | p-value |  |
| *ATXN2* expanded alleles | 0.162 | 0.150 (0.041) | **0.001** |  |
| FG (mmol/L) | 0.061 | 0.597 (0.414) | 0.154 |  |
| FI (µUI/mL) | 0.042 | -0.053 (0.053) | 0.318 |  |
| *Insulin sensitivity* |  |  |  |  |
| QUICKI | 0.029 | 2.947 (7.778) | 0.706 |  |
| Mffm/I | 0.040 | 0.110 (0.117) | 0.352 |  |
| HOMA2-%S | 0.029 | 0.002 (0.004) | 0.694 |  |
| *Insulin resistance* |  |  |  |  |
| TyG | 0.033 | -0.195 (0.591) | 0.743 |  |
| HOMA2-IR | 0.042 | -0.428 (0.437) | 0.331 |  |
| *Insulin secretion* |  |  |  |  |
| HOMA2-%β | 0.080 | -0.012 (0.006) | 0.060 |  |

FG− fasting serum glucose levels; FI− fasting serum insulin levels; TyG− Triglyceride-Glucose index; QUICKI− Quantitative Insulin Sensitivity Check Index; Mffm/I− McAuley index; HOMA2-%β− Homeostasis Model Assessment of Beta-Cell function; HOMA2-%S− Homeostasis Model Assessment of Insulin Sensitivity; HOMA2-IR− Homeostasis Model Assessment of Insulin Resistance. R^2^_adj_ – Adjusted coefficient of determination. SE – Standard error of the regression coefficient. The multiple linear models were based on the linear regression of the *ATXN2* CAG repeat length and individual glucose homeostasis parameters/indices on the INAS count. Significant associations are shown in bold.
